# Evaluation of Oil-Palm Fungal Disease Infestation with Canopy Hyperspectral Reflectance Data

**DOI:** 10.3390/s100100734

**Published:** 2010-01-20

**Authors:** Camille C. D. Lelong, Jean-Michel Roger, Simon Brégand, Fabrice Dubertret, Mathieu Lanore, Nurul A. Sitorus, Doni A. Raharjo, Jean-Pierre Caliman

**Affiliations:** 1 CIRAD, UMR TETIS, 500 Rue J.-F. Breton, 34093 Montpellier Cedex 5, France; 2 Cemagref, UMR ITAP, 361 Rue J.-F. Breton, 34196 Montpellier Cedex 5, France; E-Mail: jean-michel.roger@montpellier.cemagref.fr; 3 P.T. SMART, SMARTRI, Padang Halaban, North Sumatra, Indonesia; 4 P.T. SMART, SMARTRI, Pekanbaru, Riau, Sumatra, Indonesia; E-Mail: donie_erde@yahoo.co.id; 5 CIRAD, UR34 & P.T. SMART, SMARTRI, Pekanbaru, Riau, Sumatra, Indonesia; E-Mail: caliman@indo.net.id

**Keywords:** hyperspectral reflectance, spectroscopy, partial least square, classification, Ganoderma, oil palm

## Abstract

Fungal disease detection in perennial crops is a major issue in estate management and production. However, nowadays such diagnostics are long and difficult when only made from visual symptom observation, and very expensive and damaging when based on root or stem tissue chemical analysis. As an alternative, we propose in this study to evaluate the potential of hyperspectral reflectance data to help detecting the disease efficiently without destruction of tissues. This study focuses on the calibration of a statistical model of discrimination between several stages of Ganoderma attack on oil palm trees, based on field hyperspectral measurements at tree scale. Field protocol and measurements are first described. Then, combinations of pre-processing, partial least square regression and linear discriminant analysis are tested on about hundred samples to prove the efficiency of canopy reflectance in providing information about the plant sanitary status. A robust algorithm is thus derived, allowing classifying oil-palm in a 4-level typology, based on disease severity from healthy to critically sick stages, with a global performance close to 94%. Moreover, this model discriminates sick from healthy trees with a confidence level of almost 98%. Applications and further improvements of this experiment are finally discussed.

## 1. Introduction

**Figure 1.**
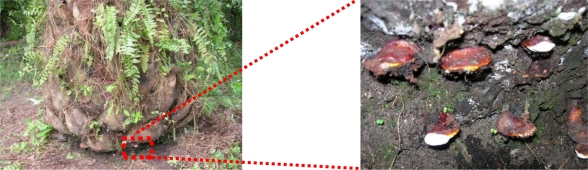
Ganoderma fruiting bodies (mushrooms) found at the bottom of sick oil-palm stem.

Early and non-destructive diagnostic of perennial crop disease is a major issue in precision farming and sustainable agriculture in general. Oil-palm plantations, in particular, strongly suffer from fungal infections (e.g., Ganoderma, like in Figure 1) but lack efficient tools to properly manage this plague without great losses in production or large use of chemicals [1].

**Figure 2.**
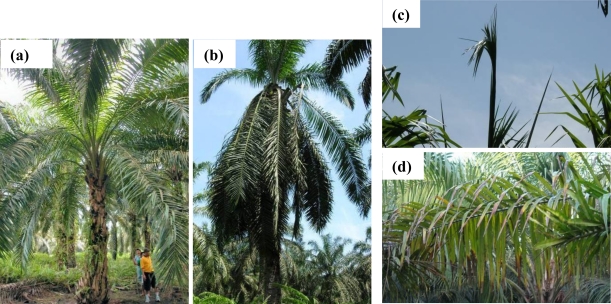
Oil palm trees in Padang Halaban Estate (Indonesia): (a) Healthy tree, (b) tree attacked by ganoderma: the lower leaves have reclined to form a skirt-like crown, (c) several unopened spears at the top of a sick tree canopy, (d) yellowing and early necrosis on leaves of a sick tree.

Ganoderma here is used as the common name for the basal stem rot induced by a fungus belonging to the genus Ganoderma, such as *Ganoderma Boninense* [2]. This disease can cause considerable damage in estates and is one of the main limitations of long-term oil-palm crop management, especially in South-East Asia. Several foliage symptoms can indicate its contamination [3] compared to a healthy tree (Figure 2a), like “skirt-like” shape of the crown due to leaves declination (Figure 2b), unopened spears (Figure 2c), and more or less yellowing and drying of the leaves (Figure 2d), that might modify the canopy properties. However, most of the time, only sampling of stem tissues and chemical analysis can allow the level of Ganoderma attack to be evaluated with confidence [4,5].

Hyperspectral reflectance spectroscopy theoretically meets the requirements of non-destructive vegetation stress detection at large scales, thanks to strong relationships between plant optical properties on one hand, and leaf pigment concentration and foliar and canopy structures on the other [6–10]. Some authors have even shown that hyperspectral data acquired by satellite or aircraft might be actually relevant to detect crop diseases or to assess crop damage severity [11–13]. However, these studies are always crop and/or disease-specific and new experiments need to be performed to validate the detectability of a different pathology in another crop. For oil palm trees in regard with Ganoderma, the modifications in the leaf or canopy optical properties due to the disease have not yet been clearly established. But they can be assumed by the theory to concern the overall canopy structure in relation to the oldest leaves declination, the unopening of new ones and the drying of others: this behaviour should be a source of reflectance change in the near infrared domain. In addition, lack of nutriments and water due to vascular circulation decrease obviously provokes yellowing and drying of the leaves, corresponding to reflectance changes in the visible domain. Even though, these influences are strongly mixed in the resulting reflectance signal, which detailed deciphering is not the scope of this paper.

We alternatively propose to detect globally the effects of the disease by means of chemometrics methods. Shafri *et al.* [14] developed a methodology based on vegetation indices and red-edge, and obtained a detection accuracy of the disease between 73% and 84%. They also tested the potential of band selection in derivative spectra to discriminate between two levels of attack, but the resulting efficiency was rather low [15]. The limitations they faced may be because the complete spectral information was only partially used, unlike in actual hyperspectral processing. Yet, there exist some robust methodologies that have proven efficient in different contexts, which could be used to classify hyperspectral spectra into several different groups, provided a good sample is available for training a statistical model of discrimination. Cluster analyses, for instance, including Linear Discriminant Analysis (LDA) allow good classifications of plant stress levels when combined with Partial Least Square Regression (PLSR) [16,17] or Principal Component Analysis (PCA) [18].

In this paper, we propose to apply such an approach to validate the efficiency of the hyperspectral reflectance spectroscopy to detect the disease, and discriminate various levels of Ganoderma fungus contamination on oil palm trees. With an objective of early remote detection and control, we evaluate different statistical models for the classification of canopy spectra into separate severity levels of Ganoderma attack.

## 2. Materials and Methods

### 2.1. Test site and ground-truth

We carried out field measurements in Padang Halaban Estate (North Sumatra, Indonesia), an oil palm plantation that has been suffering drastic attacks by Ganoderma for years. It thus includes a large range of disease severity. We have surveyed more specifically about one hundred oil palm trees, geo-localized and spotted in the plantation grid for easy subsequent identification. Each tree was assigned a score in a four-level disease typology: 0 for healthy (not sick) trees, 1 for a light attack, 2 for a medium one, and 3 for a severe (near-fatal) infestation. This classification was based on visual symptoms of the disease on the canopy and the stem (*cf.*, Table 1). Among the different trees sampled, including the healthy ones, some also showed symptoms of nutritional stress like nitrogen, iron, bore or magnesium deficiencies [19]. The ground-truth database is thus constituted of 95 samples covering a wide range of nutritional status, with 36 healthy individuals, 18 in level 1, 38 in level 2, and 3 (almost dead) in level 3.

### 2.2. Hyperspectral data

**Figure 3.**
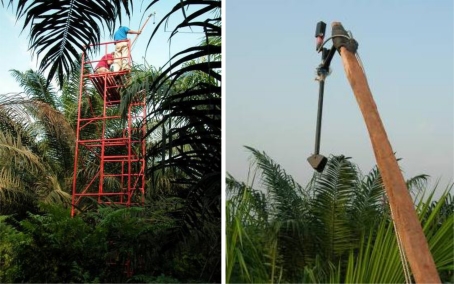
Canopy hyperspectral experiment on top of scaffoldings: a simple inertial device mounted on a shaft ensured the cosine receptor to be always looking at the zenith and canopy measurements to be acquired with the same 40° view angle.

On test site, we performed hyperspectral reflectance measurements above each of the 95 tree canopies with a Unispec spectroradiometer from PP-SYSTEMS, equipped with a Cosine Receptor and fibre optics with 20° field of view. This spectroradiometer is composed of a high precision miniature photodiode array detector that that covers 256 spectral bands in the range 310–1,130 nm, with a spectral resolution lower than 10 nm (technical information are available on http://www.ppsystems.com/Literature/EDSUniSpec-SC.pdf). Both the cosine receptor and the canopy-dedicated fibre optics were mounted on a shaft 2 m long that could be held over the canopy by an operator standing on scaffoldings (Figure 3). With a simple inertial device (of our own manufacturing), the Cosine Receptor always looked upward at zenith while the fibre optics were oriented downward towards the canopy with a view-angle of 40°. This ensured any of the radiometer acquisition comparable to others.

**Figure 4.**
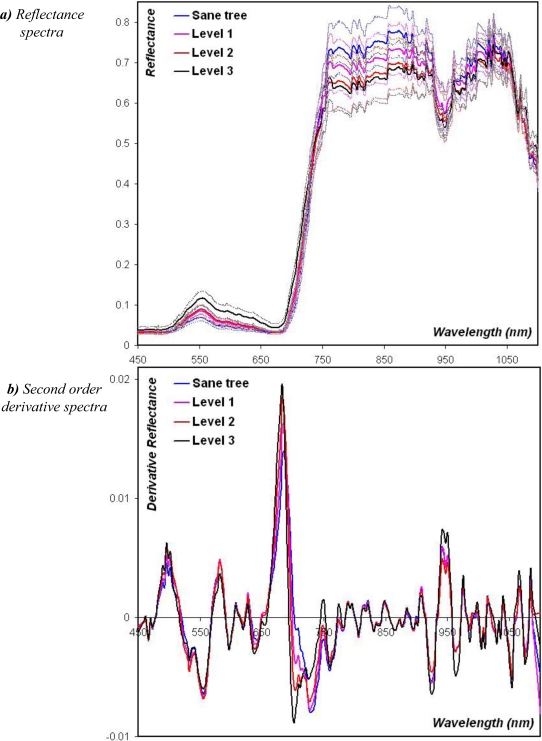
(a) Mean hyperspectral reflectance for healthy oil palms (blue) and attacked by Ganoderma at the respective levels 1 (magenta), 2 (red), and 3 (black). Faint lines indicate the contours of the envelope based on standard deviation. (b) Mean derivative filtered spectra at the 2^nd^ order of derivation for each class.

Using scaffoldings, we made tree-crown radiance measurements at 6 to 10 m height, corresponding to 1 m above the canopy. Each point thus corresponds to a measured area of about 9 m^2^ sampled on a crown area of about 60 m^2^. We made six to ten repetitions per tree to complete the whole crown. Each canopy radiance measurement was directly followed by a diffuse incident light radiance acquisition for a scaling in reflectance. Then, to limit the directional effects, we averaged these intermediate reflectance values to derive the mean reflectance of the whole tree crown. Finally, we only analyzed the spectral data acquired in the {450–1,100 nm} range because of the high level of noise in the spectra acquired in the two extreme domains {310–450 nm} and {1,100–1,130 nm} (e.g., Figure 4a); this range contains 202 spectral bands.

### 2.3. Spectra pre-processing

As spectral signatures associated with Ganoderma disease symptoms may be very faint, it is necessary to limit the signal contamination due to the variations of the sunlight and skylight illumination, the soil and other backgrounds reflectance effects, and the instrumental noise. These perturbations mainly act as baseline on the reflectance spectra, which can be as a first approximation considered as linear. The Savitzky–Golay filtering [20], that consists in a polynomial fitting followed by a derivative computation, is commonly performed to perform baseline correction [21–23].

However, the major constraint of this filter is the choice of the smoothing window size and the degree of the polynomial fit [24] along with the derivative order [21]. As no efficient methodology has been stated yet to drive this selection, we have chosen to test a large set of combinations of these parameters and extract the best solution. We thus fitted polynomials of the second and third degrees based on smoothed spectra at nine different window sizes selected to broom the spectral bins from 13 nm to 161 nm: 13, 26, 32, 39, 52, 64, 97, 129, and 161 nm. Then, we calculated their derivative function at the null, first, and second orders of derivation (e.g., Figure 4b). Derivative spectra at higher orders of derivation were not tested, following the assumption that the baselines are linear ones. It thus results in 54 different databases of derivative spectra for the sample population of 95 trees, completed by the original unprocessed reflectance data to evaluate the actual gain of preprocessing.

### 2.4. Partial Least Square Discrimination

We have applied on each of the 55 spectral databases a Partial Least Square Discriminant Analysis (PLS-DA) [25,26]. It consists in the combination of:

1. a Partial Least Square Regression (PLSR) [27,28] was calculated between the preprocessed derivative spectra and a disease degree, to transform the spectral data into uncorrelated latent variables that provides an invertible matrix for subsequent factorial discriminant analysis. Compared to data reduction such as classical band selection or vegetation index derivation, it keeps most of the initial wavelength sampling, discarding only spectral domains that provide no information, or information already contained in other domains. Figure 6 shows for instance the contribution of each wavelength to the derived PLSR components, which are significant for all the covered range except between 790 and 880 nm. To perform a simple PLSR, we chose to use it as a predictor of continuous values in the bin 0 to 1 [28,29]. Therefore, to set the disease degree, we have established an arbitrary scale suggested by a trivial linear unmixing based on the two endmembers “healthy” and “critically sick” status. We assigned values of 0 to the healthy trees, 0.4 to the trees in level 1, 0.6 to those in level 2, and 1 to those in level 3 of Ganoderma attack.
2. a Linear Discriminant Analysis (LDA) [30] was applied to the first most significant latent variables, enhancing the interclass variability while minimizing the intraclass variability of the sample to build a classification model. The selection of the number of PLSR components is guided by the compromise between minimization of Root Mean Square Error of Cross Validation (RMSECV), gain in determination coefficient (R^2^) between predicted and reference values, and stability of the model thanks to the fewer number of implied variables [31].

This PLS-DA was processed on the entire sample of 95 individuals by cross-validation [32,33] based on the “leave-one-out” method, using the “PLS” and “MASS” libraries of R-Software. It was applied independently on the 55 derived datasets. We then analysed the potential of the method to fulfil the objectives of classification of a given tree in the 4-level scoring of disease severity, comparing classification results based on the confusion matrix, and the per-class and global precisions.

## 3. Results and Discussion

The second-order derivative of a third-degree polynomial fitted on a smoothing window of 26 nm gave the best classification results. The small smoothing window size indicates that spectral features associated with the disease severity are located in small spectral ranges, showing once the great advantage of hyperspectral data compared to broadband data, inside which the Ganoderma signature might be faded out. Moreover, the high order of derivation needed to decipher the sought information is a clue of this information complexity: it argues in favor of actual hyperspectral analyses that imply all the details contained in the data, instead of the rough selection of bands and vegetation index derivations that simply crop some of those.

**Figure 5.**
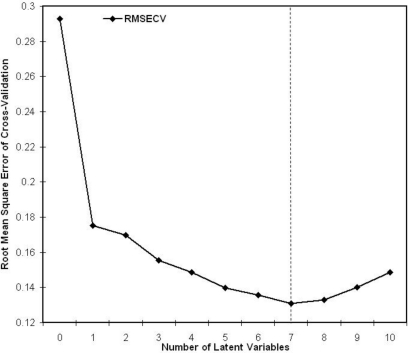
Variation of the root mean square error of cross validation (RMSECV) of models based on PLSR as a function of the number of latent variables.

**Figure 6.**
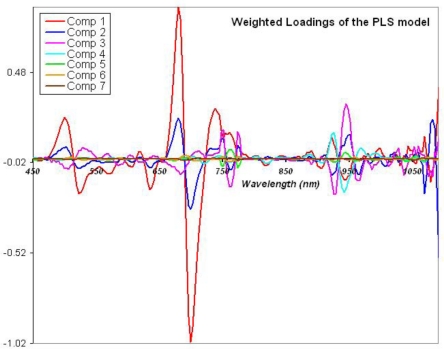
Coefficients (“loadings”) of the PLSR weighted by the corresponding eigen value for each of the seven selected components.

The determination coefficient (R^2^) and the root mean square error of cross validation (RMSECV) of possible PLSR are inversely proportional. They vary with the number of selected components, namely the latent variables of the data (Figure 5). They both present an extreme value for seven components, clearly driving our choice to this latter model. Indeed, the seven latent variables allow modelling the data with a correlation to the original spectra of 80%, which is quite robust. Let us note that the weights, or loadings, of the PLSR (*i.e.*, the coefficients of the regression) only discard the 790–880 nm domain: all the rest of the spectral range is informative, with higher weight given by the ranges 670–715 nm, 490–520 nm, 730–770 nm, and 920–970 nm (Figure 6). The 790–880 nm bin thus does not provide any valuable information for the analysis, which could be predictable based on the fact that the spectra and derivative spectra themselves that are parallel in this range (Figure 4). As almost all the spectrum is informative, it thus proves again that the complete spectral richness is needed to detect discriminating features in the canopy reflectance, compared to multispectral data in discrete and large bands.

**Figure 7.**
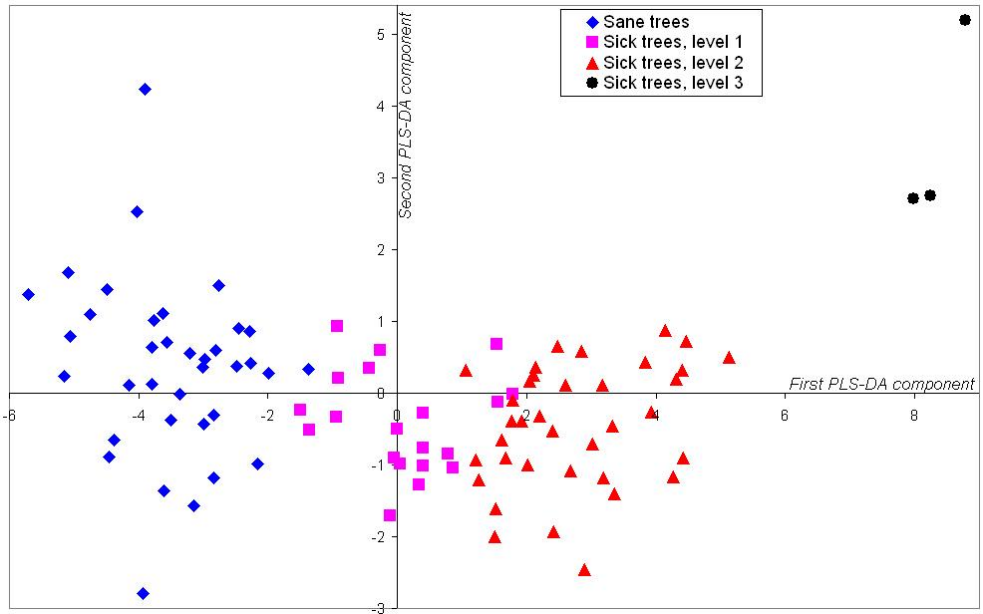
Representation of oil-palm trees in the plane defined by the two first eigenvectors of PLS-DA: healthy palms are displayed in diamond, Level 1 in square, Level 2 in triangle, and Level 3 in circle symbols.

We then applied an LDA on the scores of the resulting PLSR, which means on the new “spectra” as projected in the space described by the seven latent variables. As shown in Figure 7, displaying the oil palm trees projected in the first plane of the discriminant space, even the first discriminant factor is able to split the population into four clearly separated clusters (Figure 7). Table 2 gives the confusion matrix corresponding to the resulting LDA classification.

The resulting model thus allows a very good discrimination between the healthy and the sick trees, with only 2% of error, corresponding to “false alarm” that means that a healthy tree is found sick. In this case, results are very good because no sick tree is missed, which is the most important issue in the context of disease control. Even though, these false alarms concern only two individuals that are classified as lightly attacked by the disease; it is possible that the visual symptoms on which was based the ground-truth diagnostic were not yet observed while the reflectance spectrum already features some changes compared to healthy individuals. In this case, our field estimation of the Ganoderma level of attack was wrong while the hyperspectral reflectance analysis is already able to detect the disease. However, this should be confirmed with a sampling and chemical analysis of some stem tissues before a strong conclusion. On the other hand, these two oil palms did not show any nutritional or water deficiency that could be visually detectable and that could have been a source of modification of the reflectance independent of Ganoderma attack.

Errors occurring in the determination of disease severity only corresponds to shifts from level 1 to level 2 and inversely. Considering that the limit between these two scores for *in situ* evaluation is very fuzzy, these errors can be either due to the classification or to the field diagnosis, which is impossible to argue without a chemical proof. Even in the case of an actual classification error, these misclassifications are very few; it allows a good confidence in the overall results, presenting almost 94% of global accuracy. The cross validation process also insures the stability of the model.

The tests also show that the data pre-processing has a considerable impact on the detectability of the spectral features associated with the disease presence, and its level of severity too. As a factor of comparison, the best result obtained on the original (not filtered and not derived) reflectance spectra only gave a global accuracy of 63%, with a strong confusion between healthy and sick trees, and lots of wrong allocations of individuals inside distant classes.

The convolution of the transfer functions respectively determined by the eigenvectors of the LDA and the PLSR hence transposes the second derivative reflectance of any newly sampled tree, initially measured as a vector $\left(\begin{array}{c} R_{1}\\ \vdots \\ R_{202} \end{array}\right)$ in the space defined by the 202 wavebands, into new coordinates (*x*,*y*) such as:

$$(x,y)=\left(\begin{array}{ll} b_{1,1} & b_{1,2}\\ \vdots & \vdots \\ b_{7,1} & b_{7,2} \end{array}\right)\times \left(\begin{array}{ccc} a_{1,1} & \cdots & a_{1,5}\\ \vdots & \ddots & \vdots \\ a_{202,1} & \ldots & a_{202,7} \end{array}\right)\times \left(\begin{array}{c} R_{1}\\ \vdots \\ R_{202} \end{array}\right)$$

where a_i,j_ are the PLSR coefficients, and b_m,n_ are the LDA coefficients. The first coordinate x then allows estimating the tree degree of sickness (*cf.*, Figure 7):

- if x < −2, the tree is healthy;
- if x > 6 or 7, the tree is dramatically sick, almost dead;
- if −2 < x < 6, the threshold between Level1 and Level2 of disease severity is fuzzier and lays between 1.2 and 1.5.

It might still need some improvements to be able to fix the exact edge between Level1 and Level2, but let us remember that even in the field or in the laboratory this limit is not very well defined too.

## 4. Conclusions

Statistical algorithms like PLS-DA applied to preprocessed hyperspectral reflectance data acquired in the fields over oil palm canopies are thus efficient to detect the Ganoderma fungal disease attack with a very high confidence (∼98% of accuracy). They can even classify trees into four levels of disease severity from healthy to highly damaged with almost 94% of accuracy. Even healthy trees that present nutritional deficiencies are not misclassified as sick. It proves the potential of hyperspectral reflectance spectroscopy for oil palm crop sanitary status evaluation, using adapted processing that does not discard any spectral information by a basic spectral sampling. Indeed, results presented here show less errors and a higher performance level than those published in [14] on the basis of vegetation indices and red-edge evaluation.

In conclusion, this study clearly shows the feasibility of developing a field diagnosis tool of Ganoderma that is not destructive, and faster and cheaper than stem tissue analysis: given a tree’s canopy reflectance, it is easy to apply the PLS-DA convolution to get its new coordinate that will determine its degree of sickness. To move forward an operational tool that could be used by estate managers as a decision support, this study should be confirmed by a complementary survey including a larger sample of trees, so that the validation of the model could be performed on an independent dataset, and a stem tissue culture on specific medium to define accurately the actual status of Ganoderma attack in the tree.

It also pushes for further improvements towards remote sensing applications such as airborne or satellite-borne images analysis. Indeed, present measurements using field spectroradiometer on top of oil palm canopies is still very hard to set up and somehow dangerous, especially when dealing with mature and older trees. It might also be long to perform with a good quality. Acquiring such hyperspectral data from the air would be of major interest to cover a larger area in less time and better conditions. In addition, hyperspectral imagery would add the spatial information, and thus the opportunity to map quickly the location of attacked trees for disease control. Furthermore, focus localization and disease spreading would help analyzing the epidemiology inside a palm block, the plantation, or even the planting region depending of the width of the survey. Nevertheless, new dedicated models would then have to be calibrated for airborne or satellite-borne hyperspectral images, taking into account the imaging specificities (mainly the transfer of scales from trees to canopy).

## Figures and Tables

**Table 1.** Ganoderma-specific visual symptoms used in the fields to classified the trees into the three levels of disease severity.

**Infection degree**	**Evolution of stem conditions**	**Evolution of canopy structure**
Level 1	Presence of mycelium in the stem bark, or crumbly wood	Yellowing or drying of some leaves. One or two new leaves remain as unopened spears.
Level 2	Presence of fruiting bodies (mushrooms) at the bottom of the stem	Apparition of leaf necrosis. Three to five new leaves remain as unopened spears. Declination of older leaves.
Level 3	Rotten stem	Largely spread leaf necrosis. No new leaf. No new bunch. «Skirt-like» shape of crown due to total leaf declination.

**Table 2.** Confusion matrix obtained for the classification of oil-palm trees in four levels of disease severity.

		*Classification result*				
	Level	0	1	2	3	% of good classification
*Actual status*	0	**34**	**2**	**0**	**0**	94 %
	1	**0**	**16**	**2**	**0**	89 %
	2	**0**	**2**	**36**	**0**	95 %
	3	**0**	**0**	**0**	**3**	100 %
